# Effects of Probiotics BaSC06 on Intestinal Digestion and Absorption, Antioxidant Capacity, Microbiota Composition, and Macrophage Polarization in Pigs for Fattening

**DOI:** 10.3389/fvets.2020.570593

**Published:** 2020-11-06

**Authors:** Xuefang Cao, Li Tang, Zihan Zeng, Baikui Wang, Yuanhao Zhou, Qi Wang, Peng Zou, Weifen Li

**Affiliations:** Key Laboratory of Molecular Animal Nutrition of the Ministry of Education, Institute of Feed Science, College of Animal Sciences, Zhejiang University, and Key Laboratory of Animal Nutrition and Feed Science (Eastern of China), Ministry of Agriculture and Rural Affairs, Hangzhou, China

**Keywords:** *Bacillus amyloliquefaciens* SC06, antibiotics, intestine, antioxidant capacity, immune function, polarization, microbiota, pigs for fatteninng

## Abstract

This study aimed to compare the effects of BaSC06 and antibiotics on growth, digestive functions, antioxidant capacity, macrophage polarization, and intestinal microbiota of pigs for fattening. A total of 117 pigs for fattening with similar weight and genetic basis were divided into 3 groups: Anti group (containing 40 g/t Kitasamycin in the diet), Anti+Ba group (containing 20 g/t Kitasamycin and 0.5 × 10^8^ CFU/kg BaSC06 in the diet) and Ba group (containing 1 × 10^8^ cfu/Kg BaSC06 in the diet without any antibiotics). Each treatment was performed in three replicates with 13 pigs per replicate. Results showed that BaSC06 replacement significantly improved the ADG (*P* < 0.05), intestinal digestion and absorption function by increasing the activity of intestinal digestive enzymes and the expression of glucose transporters SGLT1 (*P* < 0.05) and small peptide transporters PEPT1 (*P* < 0.05). Besides, BaSC06 supplementation enhanced intestinal and body antioxidant capacity by activating the Nrf2/Keap1 antioxidant signaling pathway due to the increased expression of p-Nrf2 (*P* < 0.05). Notably, BaSC06 alleviated intestinal inflammation by inhibiting the production of pro-inflammatory cytokines, IL-8, IL-6, and MCP1 (*P* < 0.05), and simultaneously increasing the expression of M1 macrophage marker protein iNOS (*P* < 0.05) and M2 macrophage marker protein Arg (*P* < 0.05) in the intestinal mucosa. Moreover, BaSC06 promoted the polarization of macrophages to M2 phenotype by stimulating the STAT3 signaling pathway. It was also noted that BaSC06 improved microbiota composition by enhancing the proportion of *Firmicutes*, and reducing that of *Bacteroidetes* and *Proteobacteria*. Taken together, our results indicate that dietary supplementation of BaSC06 in pigs for fattening improves the growth, mucosal structure, antioxidative capacity, immune functions (including increasing M1 and M2 polarization of macrophage) and composition of intestinal microbiota, which is much better than antibiotics, suggesting that it is an effective alternative to antibiotics in the preparation of pig feed.

## Introduction

There is growing evidence that antibiotic resistance in humans is promoted by the extensive use of non-therapeutic antibiotics in animals leading to residues of antibiotics in animal products such as meat and milk (1). The drug resistance menace caused by the abuse of antibiotics in the animal husbandry industry has been a major societal concern in recent years. Since 2006, the European Union has banned the use of antimicrobial growth promoters in animal feed and water. Denmark, which is the world's largest exporter of pork, has further restricted the use of antibiotics to promote growth and for routine prevention of diseases (2). The Ministry of Agriculture of People's Republic of China (PRC) recently issued an “anti-drug feeding prohibition” (Announcement No. 194) explicitly prohibiting the use of feed additives containing growth-promoting antibiotics from January 1 in 2020. The antibiotics feed additive will completely disappear in the feed industry after 31 December 2020. Therefore, the most important issue facing the animal husbandry industry is the intestinal health of livestock and the quest for suitable antibiotic substitutes. Antibiotic substitutions such as probiotics, prebiotics, plant extracts, and organic acids, will be widely used in the animal husbandry industry (3, 4).

Probiotics that can improve intestinal health provide a potential alternative strategy to antibiotics feeding. Common types of probiotics include *Bifidobacterium species, Lactobacillus species, yeast, Bacillus species, Clostridium butyricum*, etc. (5). After entering the intestinal tract, these bacteria can become dominant and compete for space and nutrition with harmful bacteria in the intestine, thereby playing the role of “curing bacteria with bacteria” (6). The most commonly used probiotics in animal production are *Lactobacillus* and *Bacillus* (7–9). *Bacillus* is preferred as a feed supplement due to its higher resistance to harsh environments (10). *Bacillus amyloliquefaciens* is a probiotic strain that produces several extracellular enzymes to augment digestibility and the absorption of nutrients, as well as improving the overall immune function of the intestines (11–13). *Bacillus amyloliquefaciens* showed the potential to improve the F/G in pigs for fattening and minor porcine species given the additive at a minimum inclusion level of 1.5 × 10^8^ CFU/kg of complete feed (14). Supplementing growing pigs' diets with *B. amyloliquefaciens* (1.5 × 10^9^) CFU/kg or *B. subtilis* (1.5 × 10^9^) CFU/kg may result in improved utilization of AA and energy (15). Besides, our previous studies based on piglets have demonstrated that the probiotic BaSC06 (1 × 10^8^ CFU/kg) significantly increases growth performance (ADFI and ADG), and antioxidant status by improving digestive and absorptive enzyme activities, antioxidant capacity, and intestinal autophagy. Collectively, these enhance the intestinal integrity by improving intestinal mucosa structure, tight junctions and regulating the population of intestinal microbiota, as well as increasing immune function by activating the TLR's signaling pathway (16–18). Although BaSC06 has benefits for growth performance, comparative experiments with antibiotics remain scarce. Herein, we study the effects of BaSC06 on pigs for fattening. Besides, we speculate that BaSC06 may become a better substitute for pig fattening antibiotics. To obtain a comprehensive assessment of the intestinal physiological and biochemical situation, this research evaluated the effects of BaSC06 on the intestinal enzymatic activities for digestion and absorption, antioxidant capacity, immune function, and macrophage polarization in pigs for fattening.

## Materials and Methods

### Animals and Diets

A total of 117 pigs for fattening (Duroc × Landrace × Yorkshire) with a similar initial weight of 53 kg and a similar genetic basis were selected and randomly divided into 3 groups, each treatment was performed in three replicates with 13 pigs per replicate. Pigs for fattening in the Anti group were fed with the normal diet containing 40 g/t Kitasamycin. Pigs for fattening in the Ba group were fed the basal diet supplemented with 1 × 10^8^ CFU/kg BaSC06 without any antibiotics. Pigs for fattening in the Anti+Ba group were fed with the basal diet containing 20 g/t Kitasamycin and 0.5 × 10^8^ CFU/kg BaSC06. Because there were no pigs for fattening without antibiotics in actual production, we have not set up a negative control group. And we have to be consistent with the grouping method of the previous piglet experiments (19). All the pigs for fattening were fed *ad libitum* with basal feed and water. The adaptive period lasted 7 days, while the formal experiment period lasted 41 days, followed by sample collection and data analysis. All the experimental procedures were approved by and performed according to the guidelines of the Animal Care and Use Committee of Zhejiang University. The corn-soybean meal-based diet was supplemented with minerals and vitamins regarding to the nutritional requirements of (20). The basal diet composition and nutrition level are shown in the Table 1.

**Table 1:** Ingredient and chemical composition of the basal diet (as-fed basis).

**Ingredient**	**Content (%)**	**Nutition levels**	**Content (%)**
Corn	60.21	DE (Mcal/kg)	3.41
Soybean meal	24.55	CP (%)	17.22
Wheat bran	4.5	Lys (%)	0.84
Soybean oil	3.8	Met (%)	0.27
Corn starch	2.05	Thr (%)	0.63
Premix^a^	3	Ca (%)	0.73
Salt	0.09	Available P (%)	0.27
CaHPO4	0.9	Limestone	0.9
Total	100		

a *Premix supplied per kg: 100 mg of Fe (FeSO4), 10 mg of Cu (CuSO4), 100 mg of Zn (ZnO), 10 mg of Mn (MnO), 3086 IU of vitamin A, 386 IU of vitamin D3, 15.4 IU of vitamin E, 3.9 mg of vitamin B2, 2.3 mg of vitamin K3, 15.4 ug of B12, 15.4 ug of vitamin B5, 23 ug of vitamin B3*.

### Bacterial Strain and Kitasamycin

*Bacillus amyloliquefaciens* cells (CCTCC No: M2012280) (1 × 10^8^ CFU/g) were prepared by the Laboratory of Microbiology, Institute of Feed Sciences, Zhejiang University, China P.R. Starch was used to dilute BaSC06, and the same amount of starch was also added to every other group to compensate for the difference in the nutrient composition of the diets. Kitasamycin was obtained from Tongyi feed agriculture and animal husbandry Co., Ltd. (Qingdao, PRC).

### Sample Collection

At the end of the experiment, 2 pigs for fattening were randomly selected from each replications (*n* = 6) for sample collecting. After 24 h of fasting, the middle and posterior ileum were removed and rinsed with sterile saline, then the ileum mucosae were scraped off. Samples of ileum tissues were stored in 10% formaldehyde solution for histological observation. The intestinal mucosa and contents samples of duodenum, jejunum, ileum, and cecum were also collected. All samples were immediately frozen in liquid nitrogen and then stored at −80°C for further analysis.

### Growth Performance Assay

Weight gain (g) = [weight at the end of the experiment (kg) – weight at the beginning of the experiment (kg)]/1,000

Average daily gain (ADG) (g) = Weight gain per pig (g)/days (d)

Feed/Gain (F/G) = Feed intake per pig throughout the trial period (g)/Weight gain per pig (g).

### Hematoxylin and Eosin (H and E) Staining

The prepared paraffin sections (5 μm thick) were subjected with H&E staining and observed by a light microscope (Nikon Eclipse 80i, Tokyo, Japan). The height of the ileum villi and crypt, the thickness of the mucosa were measured with a micrometer, and the height of the villi/the depth of the crypt (V/C) was calculated.

### Biochemical Analyses

Duodenal contents digestive enzymes, which weigh about 0.5 g of duodenal contents, 0.9% normal saline was added to the appropriate volume according to the mass volume ratio of 1:9, and keep it frozen with an electric homogenizer for it to fully homogenize. The homogenate was centrifuged at 4°C and 3,500 r/min for 15 min. The protein concentration was then determined. The activities of chymotrypsin, amylase, trypsin, and lipase were determined by commercial assay kits.

Jejunum antioxidase activity: Jejunum tissue samples were taken; this is the same method as above. The kit determined the total antioxidant capacity (T-AOC), Methane dicarboxylic aldehyde (MDA), Glutathione (GSH), Catalase (CAT), Total Superoxide Dismutase (T-SOD), and Glutathione peroxidase (GSH-Px) activity.

Intestinal mucosa disaccharidase and absorptive enzyme: Appropriate jejuna mucosa samples were taken; this is the same method as above. The γ-glutamyl transpeptidase (γ-GT) kit, alkaline phosphatase (AKPase) kit and Sodium potassium ATPase (Na^+^, K^+^-ATPase) kit were utilized to assay the activity of absorptive enzymes. All the kits above were purchased from Nanjing Jiancheng Bioengineering Research Institute, and the operation method was performed according to the instructions of the kits.

### RNA Extractions and Real-Time Quantitative PCR

The total RNA isolated from intestinal mucosa (RNAiso plus, TaKaRa, Dalian, PRC) was reverse transcribed using a Prime Script II 1st Strand cDNA Synthesis Kit (TaKaRa). Quantitative and qualitative analyses of isolated RNA were assessed from the ratio of absorbance at 260 and 280 nm by NanoDrop spectrophotometer (Thermo Scientific, Wilmington, DE, USA) and agarose gel electrophoresis (Sangon Biotech, Shanghai, China). Complementary DNA (cDNA) was synthesized from l μg of total RNA using M-MLV reverse transcriptase (TaKaRa). RT-qPCR was performed according to a previous study (16) using the Premix Ex TaqTM with SYBR Green (TaKaRa), and the ABI 7500 Fast Real-Time PCR system (Applied Biosystems, Carlsbad, CA, USA). The thermocycling protocol lasted for 30 s at 95°C, followed by 40 cycles of 5 s denaturation at 95°C, 34 s annealing/extension at 60°C, and then a final melting curve analysis was made to monitor the purity of the PCR product. Primer sequences were designed and selected by Primer 5.0 and Oligo 7.0 softwares and the sequences are presented in Table S1. The 2^−ΔΔCt^ method was used to estimate mRNA abundance. Relative gene expression levels were normalized to those of the eukaryotic reference gene glyceraldehyde-3-phosphate dehydrogenase (GAPDH).

### Western Blotting

Tissue lysates were prepared using Cell Lysis Buffer for Western and IP (Biotime Biotechnology, Xiamen, Fujian, PRC). Protein concentrations were detected using a Enhanced BCA Protein Assay Kit (Biotime Biotechnology). Equal amounts of proteins from each sample were subjected to SDS-PAGE followed by a transfer of proteins to nitrocellulose membranes. Membranes were blocked with a non-protein blocking solution (Sangon Biotech) and incubated with a primary antibody overnight at 4°C. After washing with TBST, membranes were incubated with a secondary antibody linked to Horseradish peroxidase (HRP). The blots were then developed with an ECL detection system as per the manufacturer's instructions. Rabbit anti-SGLT1 (ab14686), anti-PEPT1 (ab203043), anti-P47^phox^ (ab74095), anti-Nrf2 (ab62352), anti-Keap1 (ab139729), anti-iNOS (ab178945), anti-MR (ab229987), anti-p-IRF3 (Ser396)(ab138449), and anti-IRF3 (ab25950) monoclonal antibodies were obtained from Abcam. Rabbit anti-Arg (93668S), anti-pSTAT1 (Tyr701) (9167S), anti-STAT1 (9172T), anti-STAT6 (5397S), anti-STAT3 (30835S) monoclonal antibodies were obtained from CST (Shanghai, PRC). Mouse anti-β-actin monoclonal antibody was obtained from Biotime Biotechnology. The relative band density was determined using ImageJ software.

### Cytokine Assay

Levels of Tumor Necrosis Factor-α (TNF-α), Interleukin-6 (IL-6), IL-8, IL-4, and Monocyte Chemotactic Protein 1 (MCP1) in the ileum mucosa supernatants were quantified using ELISA kits (eBioscience, Santa Clara, CA, USA) as per manufacturer's instruction.

### DNA Extraction, Pyrosequencing, and Bioinformatics Analysis

The DNA Isolation Kit (Tiangen, Beijing, China) was used for cecum DNA extraction and the quality of the extracted DNA was checked by agarose gel electrophoresis and spectrophotometric analysis. All of the genomic DNA samples were stored at −80°C for further experiments. 16s rRNA PCR amplification and 454 pyrosequencing were performed according to Qin et al. (21). Sequences obtained through 454 pyrosequencing were then filtered by QIIME software (QIIME version 1.9.1) with default parameters (22). The operational taxonomic unit (OTU) clustering pipeline UPARSE was used to select OTU at 97% similarity. Alpha diversity and beta diversity between the samples were also analyzed by QIIME software. The final taxonomic assignment was based on the consensus identification for each OTU.

### Statistical Analysis

All data were expressed as means ± SD. Results were analyzed using SPSS v16.0 software (IBM Corp.). In addition, a Student's *t*-test was used to determine differences between two groups, while one-way analysis of variance (ANOVA) followed by the Tukey's test were used for multiple comparisons. The values of *P* < 0.05 was considered to indicate a statistically significant difference. Figures were prepared using Prism 9.0 software (GraphPad Inc.).

## Results

### Substituting Kitasamycin With BaSC06 Improves Pig Growth Performance

Five indexes [Initial body weight, Final body weight, Average daily feed intake (ADFI), Average daily gain (ADG) and Feed/Gain (F/G)] were calculated for the growth performance of pig fattening. Compared with the Anti group, ADG in the Ba group significantly increased (*P* < 0.05) (Table 2), and ADFI in the Anti+Ba group and Ba group showed an upward trend though the change was not significant.

**Table 2:** Analysis of growth performance of pigs for fattening (*n* = 3).

**Items**	**Anti group**	**Anti+Ba group**	**Ba group**
Initial body weight (kg)	55.19 ± 3.22	54.97 ± 1.61	52.38 ± 2.93
Final body weight(kg)	88.19 ± 2.65	91.77 ± 3.22	88.98 ± 2.95
Average daily feed intake(g/d)	2391.11 ± 144.22	2505.37 ± 279.90	2440.59 ± 184.45
Average daily gain (g)	804.88 ± 1.32^a^	897.56 ± 3.35^b^	892.68 ± 2.91^b^
Feed/gain	2.97 ± 0.45	2.79 ± 0.24	2.73 ± 0.12

*Data were expressed as mean ± SD. In the same row, values with different small letter superscripts mean significant difference (P <0.05), and with the same or no letter superscripts mean no significant difference (P < 0.05). The following tables and figures are also consistent. Anti group = basal diet with 40g/t kitasamycin; Anti+Ba group = basal diet containing 20g/t kitasamycin and 1 × 10^8^ CFU/kg BaSC06; Ba group = basal diet containing 1 × 10^8^ CFU/kg BaSC06*.

### Effects of Substituting Antibiotics With BaSC06 on Morphology of Ileum Mucosa in Pigs for Fattening

**Figure 1:**
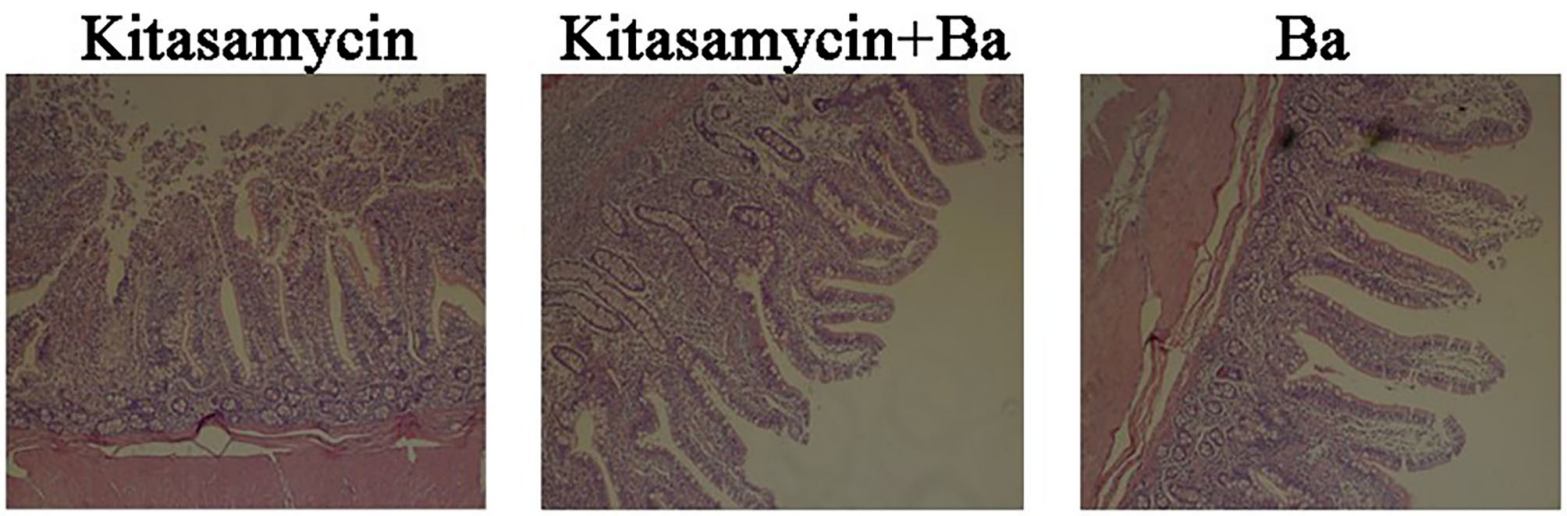
Histomorphometric analysis of ileum mucosa in pigs for fattening. H&E staining, 10 × 10 magnification. The values of villus length, crypt length and villus/crypt were calculated and shown in Table 3.

The visual effects on the morphology of ileum mucosa in pigs for fattening are shown in Figure 1. Compared with ordinary Kitasamycin treatment (Anti group), the ileum villus of the pigs for fattening in Anti+Ba and Ba groups developed better, with greater morphological integrity (Figure 1) and increased villus height (*P* < 0.05) (Table 3). However, the crypt depth in the Anti+Ba group and Ba group showed no significant change compared to the Anti group (*P* > 0.05), while V/C had an insignificant positive trend.

**Table 3:** Analysis of BaSC06 on intestinal morphology of pigs for fattening (*n* = 6).

**Items**	**Anti group**	**Anti+Ba group**	**Ba group**
Villus height (μm)	253.83 ± 2.77^a^	281.26 ± 2.44^b^	286.50 ± 1.94^b^
Crypt depth (μm)	122.08 ± 4.37	132.46 ± 3.23	134.39 ± 23.38
Villus height: crypt depth	2.08 ± 0.03	2.12 ± 0.02	2.13 ± 0.04

*The values of villus length, crypt length and villus/crypt were calculated and shown in above Table. Data were shown as mean ± SD and analyzed using one-way ANOVA Tukey test. Different letters (a, b, c) in each parameter represent significant (p < 0.05)*.

### Effects of BaSC06 on Digestion and Absorption Functions of Pigs for Fattening

#### Intestinal Digestive Enzyme Activities of Pigs for Fattening

The activity of digestive enzymes reflects the digestion and absorption capacity of feed nutrients in monogastric animals. As shown in Table 4, compared with Anti group, the trypsin, chymotrypsin, and amylase activities in the jejuna and duodena contents were dramaticaly increased (*P* < 0.05), while lipase activities were significantly decreased (*P* < 0.05) in Anti+Ba group. The trypsin and amylase activities increased significantly in Ba group only in duodena content (*P* < 0.05), while chymotrypsin and lipase activities decreased remarkably in both intestinal contents by comparison with those in Anti group (*P* < 0.05).

**Table 4:** Analysis of the digestive enzyme activities in intestinal contents of pigs for fattening (*n* = 6).

**Items**	**Anti group**	**Anti+Ba group**	**Ba group**
**Duodenum**			
Trypsin (U/mgprot)	261.60 ± 25.61^b^	507.72 ± 3.91^a^	465.38 ± 26.99^a^
Chymotrypsin (U/gprot)	3594.01 ± 384.10^b^	6074.17 ± 597.89^a^	2718.31 ± 128.35^b^
Amylase (U/mgprot)	519.10 ± 25.81^b^	746.66 ± 46.52^a^	861.53 ± 26.28^a^
Lipase (U/gprot)	22.92 ± 2.08^a^	14.37 ± 0.04^b^	14.56 ± 5.40^b^
**Jejunum**			
Trypsin (U/mgprot)	576.80 ± 63.71^b^	1022.91 ± 39.52^a^	580.42 ± 33.62^b^
Chymotrypsin (U/gprot)	6334.65 ± 580.79^a^	4155.19 ± 369.86^b^	2154.20 ± 223.70^c^
Amylase (U/mgprot)	767.73 ± 20.49^b^	1221.74 ± 19.75^a^	744.73 ± 24.71^b^
Lipase (U/gprot)	159.20 ± 7.45^a^	60.30 ± 4.55^b^	29.81 ± 3.51^c^

*Data were analyzed by one-way ANOVA Tukey test. Different letters (a, b, c) in each parameter represent significant (p < 0.05)*.

#### Disaccharide Enzyme Activity in Jejunal Mucosa of Pigs for Fattening

As shown in Table 5, BaSC06 can enhance the enzymatic activity of the mucosal disaccharide in jejuna. Compared with the Anti group and Anti+Ba group, the sucrase in the Ba group significantly increased by 66.4% (*P* < 0.05) and 39.8% (*P* < 0.05), lactase activities increased by 100% (*P* < 0.05) and 105.7% (*P* < 0.05), respectively. However, three disaccharide enzymes (sucrase, maltase, and lactase) in the Anti+Ba group were not significantly affected.

**Table 5:** Analysis of the disaccharidase activities in jejunum mucosa of pigs for fattening (*n* = 6).

**Items**	**Anti group**	**Anti+Ba group**	**Ba group**
Sucrase (U/mgprot)	86.65 ± 1.52^b^	103.14 ± 10.23^b^	144.16 ± 3.14^a^
Maltase (U/mgprot)	209.94 ± 8.53	196.47 ± 22.69	179.89 ± 11.78
Lactase (U/mgprot)	1.09 ± 0.10^b^	1.06 ± 0.19^b^	2.18 ± 0.12^a^

*Data were analyzed by one-way ANOVA Tukey test. Different letters (a, b, c) in each parameter represent significant (p < 0.05)*.

#### Enzyme Activities Related to Absorption in Jejunal Mucosa of the Pigs for Fattening

Compared with the Anti group, half substitution of Kitasamycin with BaSC06 could significantly spark activities of AKPase and Na^+^, K^+^-ATPase in jejuna mucosa by 47.3 and 47.7%, respectively, while complete substitution only increased Na^+^, K^+^-ATPase activity by 76.6% and dramatically increased γ-GT activity (*P* < 0.05) (Table 6).

**Table 6:** Analysis of the absorptive enzyme activities in jejunum mucosa of pigs for fattening (*n* = 6).

**Items**	**Anti group**	**Anti+Ba group**	**Ba group**
γ-Glutamytransferase (U/gprot)	40.58 ± 1.15^a^	37.78 ± 2.94^a^	25.41 ± 2.48^b^
AKPase (Kingunit /gprot)	748.25 ± 6.56^b^	1102.10 ± 57.66^a^	679.67 ± 19.33^b^
Na^+^, K^+^-ATPase (U/mgprot)	3.50 ± 0.23^b^	5.17 ± 0.23^a^	6.18 ± 0.50^a^

*Data were analyzed by one-way ANOVA Tukey test. Different letters (a, b, c) in each parameter represent significant (p < 0.05)*.

#### Substituting Kitasamycin With BaSC06 Increases the Expression of Transporters

**Figure 2:**
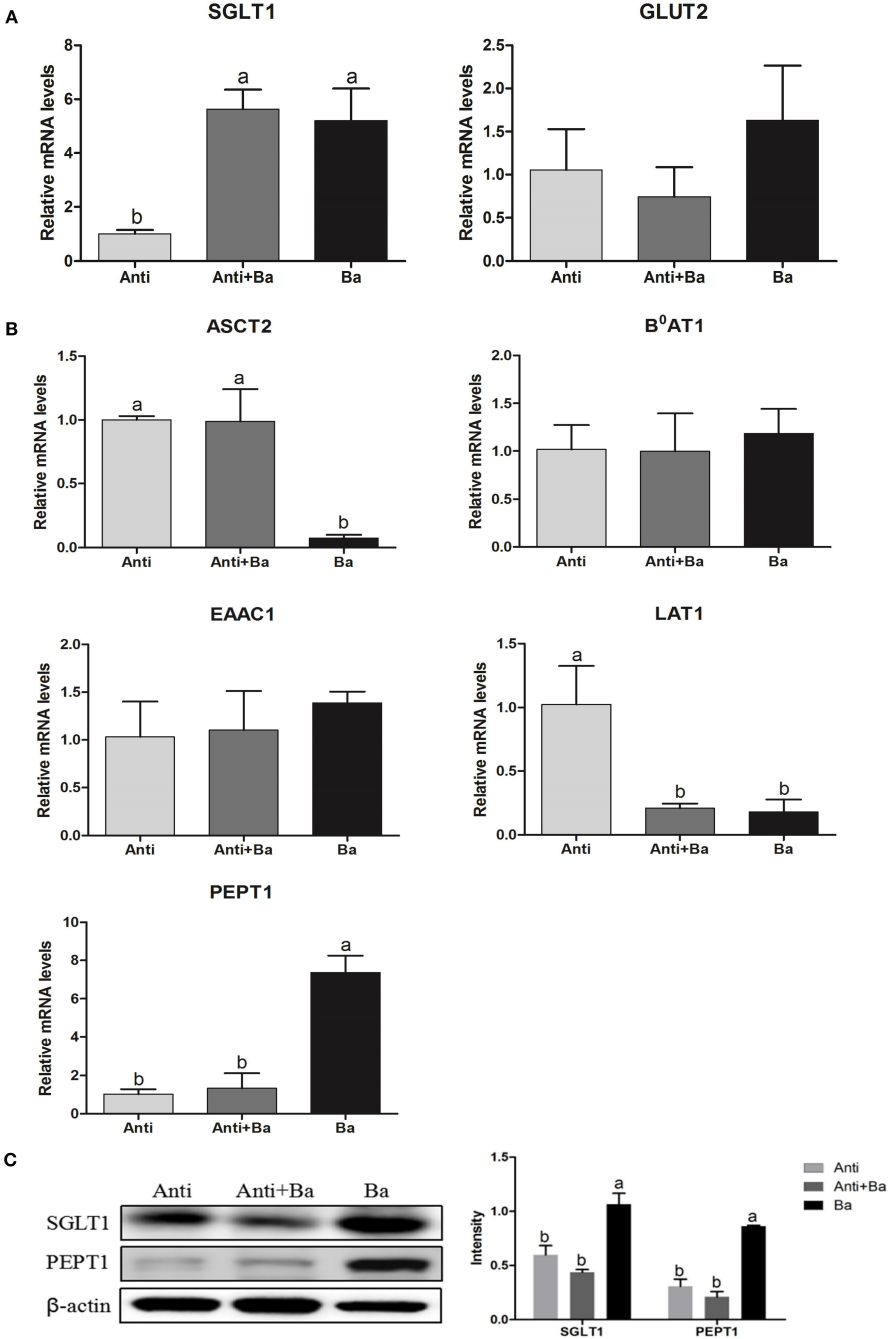
BaSC06 could increase the expression of glucose transporters and small peptide transporters in jejunum mucosa of pigs for fattening (*n* = 6). **(A,B)** The mRNA levels expression of SGLT1, GLUT1, ASCT2, B^0^AT1, EAAC1, LAT1, and PEPT1 in jejunum of pigs for fattening were analysis with RT-qPCR, the 2^−ΔΔCt^ method was used to estimate mRNA abundance. Different letters (a, b, c) in each parameter represent significant (*p* < 0.05). **(C)** Protein lysates from jejunal mucosa were examined by western blot for SGLT1 and PEPT1 levels. The ratio of SGLT1 and PEPT1 to β-actin were analyzed using ImageJ. Different letters (a, b, c) in each parameter represent significant (*p* < 0.05).

Compared with the Anti group, BaSC06 administration significantly increased the expression of the glucose transporter sodium-dependent glucose transporters 1(*SGLT1*) in the jejunum by 78.3% (*P* < 0.05), whereas, there was no significant difference in the mRNA expression level of the glucose transporter 2 (*GLUT2*) in the Ba group and the Anti+Ba group (*P* < 0.05) (Figure 2A). Results from the expression of four amino acid transporters revealed that BaSC06 could markedly regulate mRNA expression levels of the neutral amino acid transporter 2 *(ASCT2)*, cationic amino acid transporter and L-type amino acid transporter 1 *(LAT1)* in the jejuna mucosa. Compared with the Anti group, the expression of LAT1 mRNA decreased significantly in Ba group and Anti+Ba group (P < 0.05). Whereas, the level of *ASCT1* mRNA expression only decreasd significantly in Ba group compared to Anti group (*P* < 0.05). In addition, the level of small peptide transporter 1 (*PEPT1*) mRNA expression only increased significantly in Ba group compared to Anti group (*P* < 0.05) (Figure 2B). The expression levels of SGLT1 and PEPT1 were further verified by results of Western blotting. Compared with the Anti group, feed supplemented with BaSC06 alone significantly elevated the protein expression levels of Na^+^-dependent glucose transporter SGLT1 and small peptide transporter PEPT1 in the jejunum by 78.3% (*P* < 0.05) and 177.4%, respectively (*P* < 0.05) (Figure 2C).

### Antioxidant Profiles of Pigs for Fattening

#### BaSC06 Increases Intestinal Antioxidant Enzyme Activities

**Figure 3:**
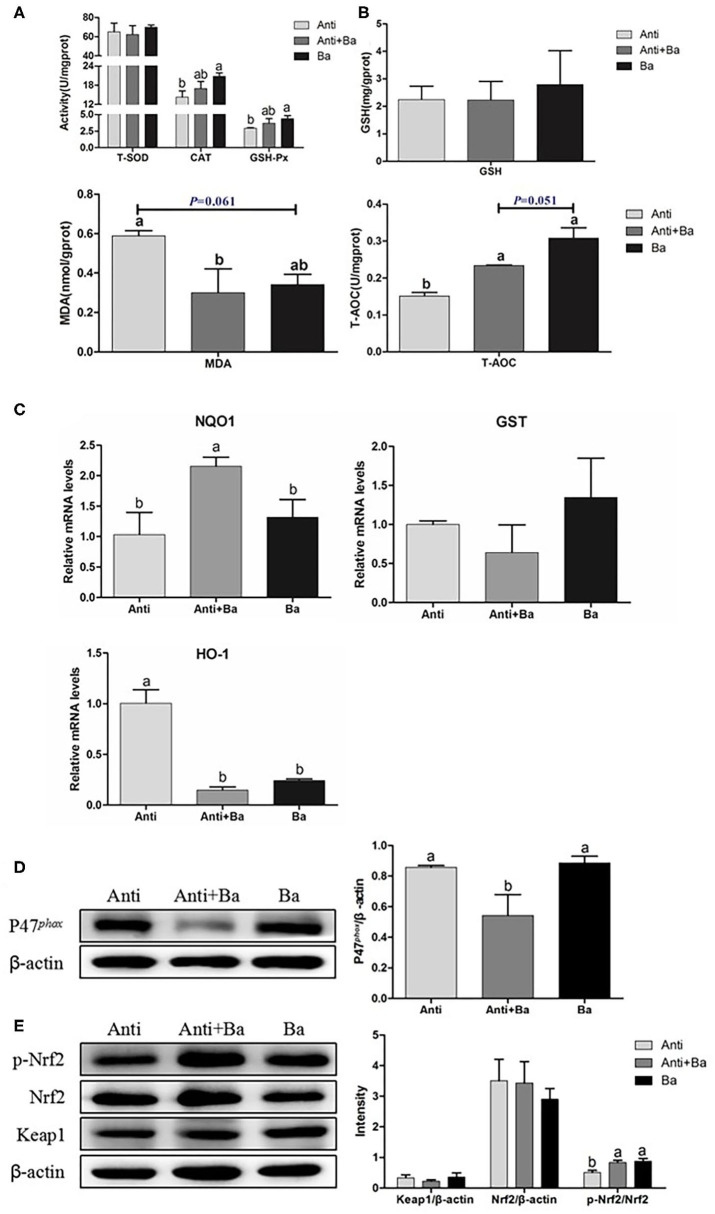
BaSC06 enhanced antioxidant properties and Nrf2/Keap1 signaling pathway in pigs for fattening jejunum (*n* = 6). **(A,B)** The antioxidant capacity was determined by measuring GSH-Px, T-SOD, CAT, GSH, MDA, and T-AOC. Data are presented as the mean ± SD, one-way ANOVA with Tukey test. **(C)** The mRNA levels expression of genes related to anti-oxidation in jejunum of pigs for fattening were analysis with RT-qPCR, the 2^−ΔΔCt^ method was used to estimate mRNA abundance. **(D,E)** Protein lysates from jejunal mucosa were examined by western blot for P47^phox^, p-Nrf2, Nrf2, Keap1 levels. The ratio of P47^phox^, p-Nrf2, Nrf2, Keap1 to β-actin were analyzed using ImageJ. Different letters (a, b, c) in each parameter represent significant (*p* < 0.05).

BaSC06 supplement could reduce the MDA level in the intestine by 48.9% (*P* < 0.05) and 42.0% (*P* = 0.061) for the Anti+Ba and Ba groups, respectively (Figure 3B). At the same time, the BaSC06 supplement could significantly improve the intestinal T-AOC content (*P* < 0.05) (Figure 3B). Compared to the Anti group, CAT and GSH-Px activities in the Anti+Ba group showed no significant change (*P* > 0.05) (Figure 3A), but increased by 45.4% (*P* < 0.05) and 49.4% (*P* < 0.05) in Ba group, respectively. There was no significant difference between the Anti+Ba group and Ba group in T-SOD activity and GSH level (*P* > 0.05) (Figure 3A).

#### Antioxidant-Related Genes mRNA Expression in Jejunum of Pigs for Fattening

The expression of antioxidant-related genes is also an important indicator for determining the body's antioxidant capacity. Compared with the control group, antibiotics substituted with BaSC06 significantly increased the relative gene expression levels of NAD (P)H and Quinone oxidoreductase 1(*NQO1*), while Anti+Ba group significantly increased the expression of the NQO1 gene by 108.5% (*P* < 0.05) (Figure 3C). The HO-1 gene expression in the Anti+Ba group as well as the Ba group decreased significantly by 85.3 and 76.1% respectively (*P* < 0.05) (Figure 3C). There was no significant difference in the expression of glutathione S-transferase (*GST*) mRNA among the three groups (*P* > 0.05) (Figure 3C).

#### Substituting Kitasamycin With BaSC06 Activated Nrf2/Keap1 Signaling Pathway in Jejuna of Pigs for Fattening

Compared to the Anti group, the relative expression of P47^phox^ protein in the jejunal mucosa in Anti+Ba group was significantly reduced by 36.8% (*P* < 0.05) (Figure 3D), there was no significant difference between the Ba and Anti groups (*P* > 0.05) (Figure 3D). the phosphorylated Nuclear transcription factor-erythroid 2-related factor (Nrf2) proteins in the jejunum of the Anti+Ba group and Ba group were significantly up-regulated by 64.9% (*P* < 0.05) and 74.3% (*P* < 0.05) (Figure 3E). However, the relative expression levels of Kelch-like epichlorohydrin-associated protein 1 (Keap1) and Nrf2 proteins did not vary significantly (*P* > 0.05) (Figure 3E).

### Immune Function of Pigs for Fattening

#### Immune Cytokines Expression in Ileum Mucosa of Pigs for Fattening

**Figure 4:**
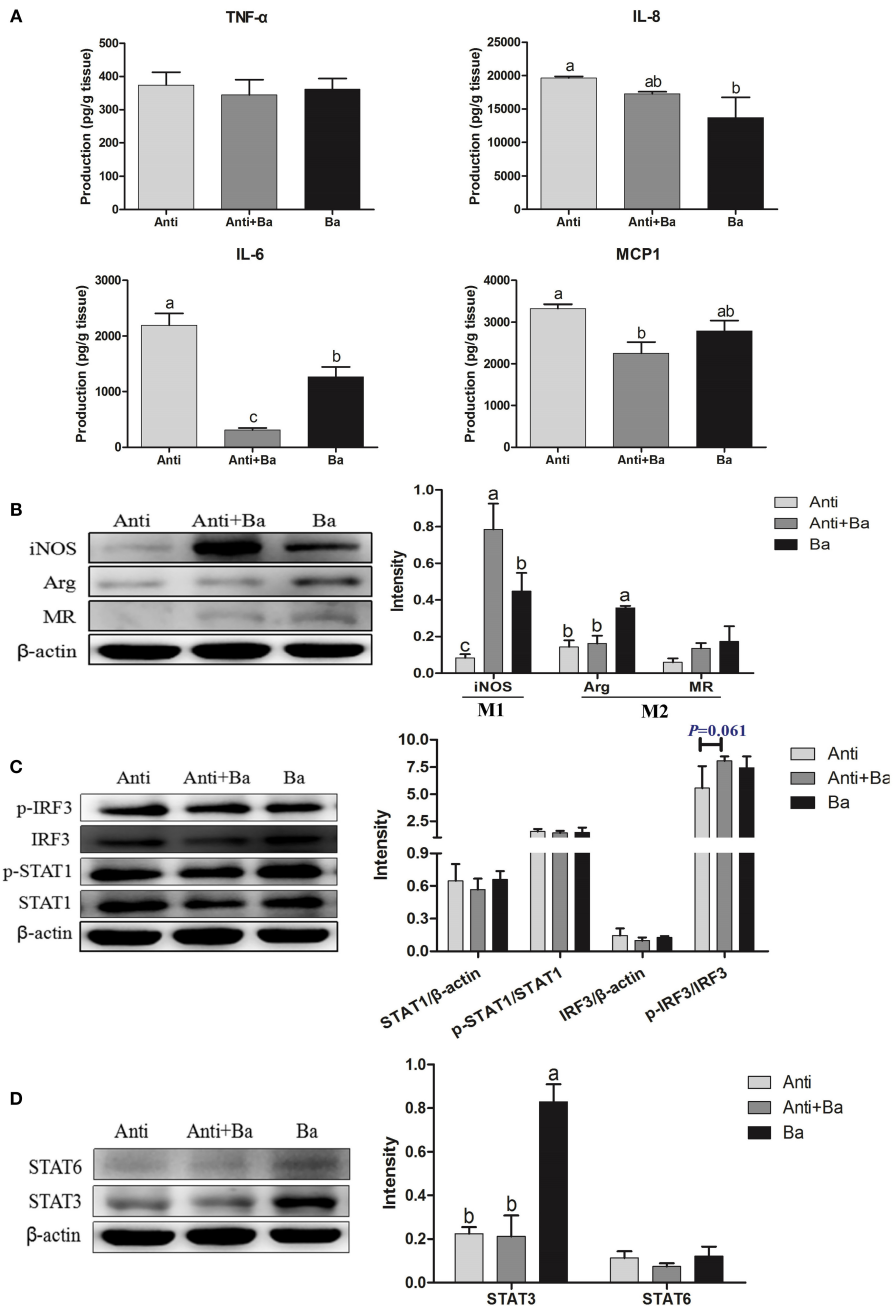
Effects of BaSC06 on Immune Function (*n* = 6). **(A)** The Production of Immune Cytokines in ileum of pigs for fattening was determined by measuring TNF-α, IL-8, IL-6, and MCP1 using ELISA. Data are presented as the mean ± SD, one-way ANOVA with Tukey test. Different letters (a, b, c) in each parameter represent significant (*p* < 0.05). **(B–D)** Protein levels of macrophage polarized markers iNOS, Arg and MR, as well as key protein of polarization related signaling pathway p-IRF3, IRF3, p-STAT1, STAT1, STAT6, and STAT3 were analyzed using western blotting. The ratio of iNOS, Arg, MR, STAT1, IRF3, STAT3, and STAT6 to β-actin, p-STAT1 to STAT1, and p-IRF3 to IRF3 were analyzed using ImageJ. Different letters (a, b, c) in each parameter represent significant (*p* < 0.05).

The level of pro-inflammatory cytokines IL-6 and chemokine MCP1 in the ileum mucosa in Anti+Ba group was significantly reduced by 86.0% (*P* < 0.05) and 32.3%, respectively (*P* < 0.05) (Figure 4A), while the levels of IL-6 and IL-8 in the Ba group were significantly reduced by 42.3% (*P* < 0.05) and 13.6% (*P* < 0.05), respectively (Figure 4A). However, significant increased IL-6 was found in the Ba group compared with the Anti+Ba group (*P* < 0.05) (Figure 4A).

#### Effects of BaSC06 on Expression of Key Proteins in M1/M2 Macrophages Polarization Signaling Pathways

The expression of inducible nitric oxide synthase (iNOS), a marker protein of M1 polarized macrophages, increased significantly in the two groups supplemented with BaSC06 (*P* < 0.05) (Figure 4B), indicating that BaSC06 may promote M1 polarization of intestinal macrophages. What's more, the expression of Arginase (Arg), a marker protein of M2 macrophages, was also significantly up-regulated in the Ba group compared with the Anti group and the Anti+Ba group (*P* < 0.05) (Figure 4B). The expression of Mannose receptor (MR) (the another M2 polarized marker protein) among three groups remained unchanged (*P* > 0.05) (Figure 4B).

There were no significant changes in the signal transduction and expression of activators of the transcription signal transducer and activator of transcription (STAT1), p-STAT1, and the interferon regulator IFN-regulatory factor 3 (IRF3) in BaSC06 supplemented feed (*P* > 0.05) (Figure 4C). The upward trend revealed by the expression of phosphorylated IRF3 protein in the Anti+Ba group was however not significant (*P* = 0.061). The expression of the STAT3 protein in the Ba group increased significantly (*P* < 0.05), whereas STAT6 showed no change (*P* > 0.05) (Figure 4D).

### Substituting Antibiotics With BaSC06 Affects the Composition of Intestinal Microbiota

**Figure 5:**
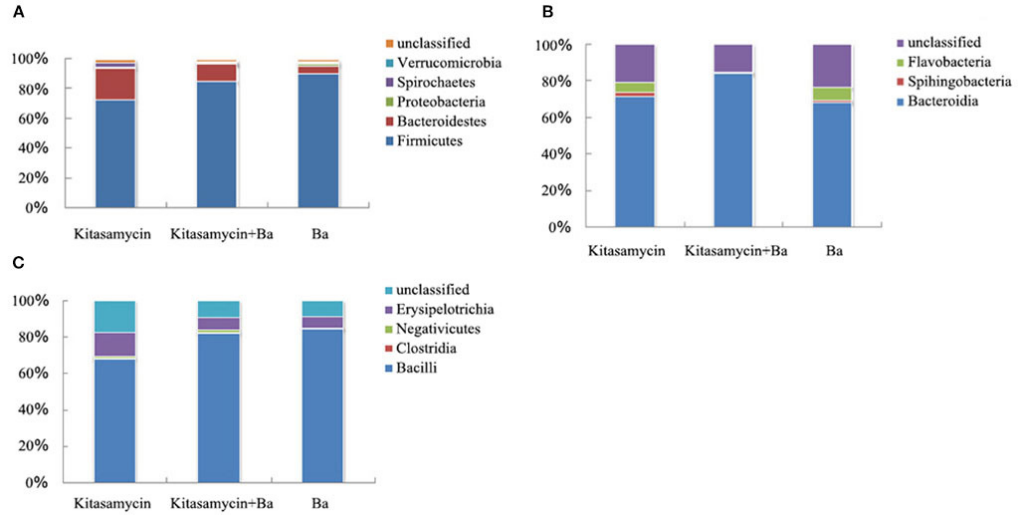
BaSC06 changes the composition of intestinal microbiota without affecting the diversity of intestinal microbiota of pigs for fattening (*n* = 3). **(A)** Phylum classification statistical chart of flora in cecum contents of pigs for fattening. **(B)** Phylum classification statistical chart of Bacteroidetes in cecum contents of pigs for fattening. **(C)** Phylum classification statistical chart of Firmicutes in cecum contents of pigs for fattening.

In addition to the unclassified bacteria, there are five main categories of *Verrucomicroba, Spirochaetes, Proteobacteria, Bacteroidetes, and Firmicutes* in the cecum flora of pigs for fattening (Figure 5A). At the Class level, we further analyzed the effect of BaSC06 probiotics on *Bacteroides* and *Flavobacteria* in *Bacteroidetes* (Figure 5B). *Bacteroides* accounted for the vast majority of *Bacteroidetes*, 71.09, 83.50, and 67.66% in Anti group, Anti+Ba group and Ba group, respectively, while the amount of *Flavobactera* was quite low (Figure 5B). *Firmicutes* in the caecum of pigs for fattening could be divided into *Bacilli, Clostridia* and *Erysipelotrichia*. *Bacilli* accounted for 67.98, 82.13, and 84.48% of *Firmicutes*, respectively in three groups (Figure 5C). *Erysipelotrichi* accounted for 13.9, 6.77, and 6.15% of *Firmicutes*, respectively (Figure 5C).

## Discussion

The problem of residual antibiotics and drug resistance caused by the abuse of antibiotics have aroused widespread concern. As a green feed additive with the potential to replace antibiotics (23), probiotics are widely promoted due to their ability to significantly improve the efficiency of production and the welfare of the animal husbandry industry (24). Instead of aureomycin, our previous study found that BaSC06 could increase the growth performance of piglets by improving ADG (19). In the current study, BaSC06 administration could also improve the growth performance of pigs for fattening, which is related to improving intestinal health, including digestion absorption function, antioxidant capacity, immune function, and microbiota composition (25–28). Although the supplementation of 0.2% probiotics *Bacillus* (including *Bacillus subtilis, Bacillus coagulans*, and *Lactobacillus acidophilus*) in the diet could significantly increase the ADG of pigs for fattening, it has no significant effect on the ADFI and F/G (29). Balasubramanian et al. found that the *Bacillus* compound not only increased the ADG significantly but also improved F/G (30).

Digestive enzyme activity is a reflection of the digestion and absorption capacity of feed nutrients in monogastric animals. A large number of di-and tripeptides are also generated when the protein is hydrolyzed in the intestine to produce free amino acids, which are the main forms of protein absorption (31). γ-Glutamytransferase can regulate the glutamine nutritional metabolism of the intestinal epithelial cells, improve the absorption, transport, and utilization of amino acids by cells, thus maintaining the integrity of the intestinal mucosa, improving intestinal health, and creating favorable digestion and absorption conditions (32, 33). We found that BaSC06 alone significantly reduced the activity of γ-Glutamytransferase. This may be due to the fact that BaSC06 can enhance the absorption capacity of the cell, resulting in reduced demand for γ-Glutamytransferase. Moreover, our research found that BaSC06 could increase activities of trypsin, amylase, disaccharase (sucrase and lactase) and Na^+^/K^+^-ATPase in the pigs' intestine for fattening, thereby promoting the digestion and utilization of protein and carbohydrates in the diet. Several studies have reported that *Brevibacillus brevis FJAT-1501-BPA* or *Bacillus subtilis* supplementary significantly improved the feed conversion rate and the growth performance of weaning piglets by regulating trypsin and amylase (34, 35). Goyal et al. have found that feeding rats with *Lactobacillus* La1 increased sucrase activity in the intestine. Besides, pre-oral administration of LGG increased sucrase and lactase activity (36). Na^+^/K^+^-ATPase can hydrolyze ATP to help the body obtain energy, transport Na^+^ against chemical gradients, and regulate the electrochemical balance of Na^+^ and K^+^ inside and outside the intestinal cell membrane. We speculate that the changes in the digestive enzyme activity observed in this study could be attributed to probiotic secretion and the stimulation of endogenous enzyme synthesis in intestines (37).

Transporters participate in the transmembrane transport of glucose, di- and tripeptides. SGLT1 (Sodium-dependent glucose transporter 1) and GLUT2 (Glucose transporter 2) mediate the transport of glucose molecules across the intestinal epithelium brush border (38, 39). There are at least five kinds of small peptide transporters, and PEPT1 and PEPT2 are the most widely studied. PEPT1 is highly expressed in the brush border of the jejunum epithelium of pigs and mediates intestinal absorption of di- and tripeptides (40, 41). Interestingly, we found that dietary supplementation of BaSC06 alone could increase the expression of SGLT1 and PEPT1. Furthermore, Na^+^/K^+^-ATPase is particularly important for maintaining the distribution of Na^+^-dependent transporters such as SGLT1 (Sodium-dependent glucose transporter 1) and GLUT2 (Glucose transporter 2) in the small intestine epithelium. Therefore, the increased Na^+^/K^+^-ATPase activity and expression of SGLT1 and PEPT1 implied that BaSC06 could improve the absorption of glucose and peptide, thereby influencing the metabolism of carbohydrates and proteins.

Oxidative stress causes damage to the intestinal mucosal cells and intestinal tissues of animals, as well as metabolic disorders in the body, which could induce a series of intestinal diseases leading to abnormal intestinal functions (42–44). The levels of oxidation and reduction in the body normally maintain a dynamic balance but can be broken by a variety of harmful endogenous and exogenous stimuli. If the levels of free radicals and ROS exceed the reduction level of the antioxidant system, the body's antioxidant capacity decreases, resulting in oxidative damage to biological macromolecules (45). Free radicals can cause peroxidative damage to cell membrane lipids and produce MDA that attacks polyunsaturated fatty acids in biological cell membranes, further triggering lipid peroxidation to cause more severe oxidative stress damage (46). The main antioxidant defense machinery consists of enzymes (CAT, SOD, GSH-Px, etc.) and biological antioxidants (47). In addition to antioxidant enzymes, essential phase II detoxifying enzymes such as NQO1, GST, HO-1, and γ-GCS may also play an antioxidant role in the antioxidant process. NQO1 can catalyze reductions in quinone compounds and prevent unstable semiquinones from forming. Therefore, it can protect cells from oxidative damage caused by heterologous biological substances such as quinones (48). HO-1 is an inducible phase II detoxifying enzyme (49) the expression of which is regulated by Nrf2 (50), and its increased expression may also be utilized as an adaptive mechanism for cell self-protection under oxidative stress (51, 52). Wang et al. found that HO-1 is a sensitive index for oxidative stress (16). The decrease of HO-1 mediated by BaSC06 indicates a reduction in oxidative stress. Several studies have demonstrated excellent antioxidant activities in probiotics. For instance, *Lactobacillus casei* significantly reduced MDA content in pig serum and relieved Lipopolysaccharide (LPS)-induced oxidative stress (53). In our study, BaSC06 reduced the MDA content in the intestinal mucosa of pigs for fattening by increasing gene expression of CAT, GSH-Px, and NQO1, indicating that the strain induces good antioxidant activity and prevents oxidative stress damage to the intestinal mucosa. These results are consistent with our group's previous research on BaSC06 in piglets and rats (19, 54).

The Nrf2/Keap1 signaling pathway is important for the body to resist oxidative stress (55, 56). Recent studies elucidated the mechanism by which microorganisms activate the Nrf2 signaling pathway, protect the intestinal epithelium from oxidative stress and other endogenous stimuli, and maintain the dynamic balance and self-protection of intestinal epithelial cells (57–59). Chowdhury et al. found that *Bacillus megaterium RB-05* up-regulates the translation and accumulation of Nrf2 and Keap1 by secreting low fructose in its nuclear to stimulate the expression of downstream transcription factors like HMOX1, NQO1, GSTA2, SOD1, and GPX1 (60). We found that BaSC06 increased the expression of phosphorylated Nrf2 protein in the jejunal mucosa of pigs for fattening. However, it did not influence the expression of Nrf2 and Keap1, which is similar to the results on intestinal porcine epithelial cells-1 (IPEC-1) cell experiments (16). Therefore, it could be concluded that the activation of the Nrf2/Keap1 signaling pathway, indicated by higher p-Nrf2 protein expression in the jejunal mucosa of pigs for fattening induced by BaSC06, could promote Nrf2 nuclear translocation. Transcription and translation of downstream genes (such as the aforementioned antioxidase GSH-Px and CAT, phase II detoxifying enzymes, etc.) then resulting in the improvement of the total intestinal antioxidant capacity, the protection of the intestine from oxidative damage, the maintenance of the normal physiological function of the intestine, and ultimately the promotion of digestion and absorption. These results are similar to previous studies on other probiotics with antioxidants (57, 61).

Besides antioxidant enzymes, probiotics can also reduce oxidative stress by reducing ROS production. The main source of reactive oxygen species *in vitro* is the reactive nicotinamide adenine dinucleotide phosphate oxidase (NOX). P47^phox^ is considered a key regulator of NOX activity (62). Probiotics alter NOX activity by regulating the expression of P47^phox^. Rashid et al. showed that oral administration of probiotics could protect rats from vascular endothelial dysfunction induced by common bile duct infarction, via the regulation of P47^phox^ protein expression (63). Additionally, Tapia-Paniagua et al. suggested that the probiotic SpPdp11 down-regulates the transcription of NOX in *Solea senegalensis* (64). In our previous study, BaSC06 replacement did not affect NOX activity and the P47^phox^ expression in piglets (19). However, we found that BaSC06 alone could inhibit the production of the excess ROS by down-regulating the expression of the NOX active subunit P47^phox^ protein in fattening pigs, which is consistent with our present results on the results in IPEC-1 (16).

The intestinal mucosa, lymphocytes, plasma cells, and macrophages contain abundant lymphoid tissues that respond to harmful gut antigens such as food toxins, bacteria, and viruses. Therefore, intestines are the first immune defense system of the digestive system, otherwise known as the intestinal mucosa immunity (65). The interaction between microorganisms and intestinal epithelial cells is the beginning of the host immune response, which can eliminate potential pathogenic microorganisms (66, 67). Drouaultholowacz et al. found that a probiotic with an anti-inflammatory effect can effectively prevent inflammation of the intestine by increasing the level of anti-inflammatory factors and reducing the level of pro-inflammatory factors (68). *In vivo*, IL-6 and IL-8 levels in the intestinal mucosa of piglets decreased significantly when *Enterococcus faecium* was added to the diet (69). It has been reported that LGG can effectively reduce the level of pro-inflammatory factors in the intestinal mucosa of mice, increase the level of anti-inflammatory factors, and enhance the immune response of flagellate infected mice (70). *In vitro* co-culture of *Lactobacillus reuteri* and IPEC-1 was found to significantly inhibit TNF-α and IL-6 overexpression induced by *Escherichia coli K88* or lipopolysaccharide, and to significantly promote the expression of IL-10 (71). Our study also revealed that BaSC06 inhibits intestinal inflammation in pigs for fattening by regulating the expression of pro-inflammatory cytokines IL-6, IL-8, and MCP1 in the intestinal mucosa.

As innate immune cells in the gut, macrophages play a key role in maintaining intestinal homeostasis, regulating cytokine secretion and generating immune response (72–74). Macrophages can sense the stimulation from microorganisms and activate polarization. However, polarization differences of M1 or M2 macrophage types are determined by the diversity of microorganisms present (75–77). Classical activation of M1 macrophages in response to IFN-γ is characterized by a high ability to express antigens, high expressions of iNOS, IL-6, TNF-α, IL-1β mRNA, and nitric oxide (NO), which can kill endogenous pathogens and tumor cells (78). In contrast, the activation of M2 macrophages is promoted by various factors (such as IL-4), which is consistent with the high mRNA expression of Arg1, Fizz1, Ym1, and MR, playing an important role in the inflammatory response, debris removal, blood vessels generation as well as tissue repair and reconstruction (79). The polarization imbalance of macrophages i.e., over enhancement of M1 polarization damages immunity, while the over enhancement of M2 polarization promotes chronic infection, thereby inducing various diseases (80). Fu et al. reported that BaSC06 directly induces M1 phenotype polarization, and M2 phenotype polarization via microbiota modification (81). For instance, *Bacillus amyloliquefaciens* enhanced phagocytic bactericidal functions by inducing M1 polarization of macrophages derived from the mouse bone marrow (82). In this study, BaSC06 could simultaneously up-regulate the expression of M1 (iNOS) and M2 (Arg) macrophage marker proteins thereby promoting cell phagocytosis and sterilization, and enhancing cell anti-inflammatory and anti-infection abilities. Therefore, we speculate that BaSC06 could maintain the intestinal immune balance of pigs for fattening. Our current results of intestinal mucosal cytokines showed that BaSC06 enhanced the immune tolerance of intestinal mucosa in pigs for fattening and inhibited inflammation of the intestinal mucosa. Accordingly, the expression of macrophage polarization marker protein may adapt to this change in the microenvironment, avoid the extreme situation of M2 type immune tolerance, maintain the M1 and M2 polarization balance, and promote pro- and anti-inflammatory balance to maintain intestinal homeostasis. Studies have shown that STAT3 inhibition contributes to the polarization of the macrophages toward the M1 phenotype (83). It has been reported that the increase of M1 phenotype is related to the activation of p38 (84, 85). In the current study, the expression of STAT3 protein in BaSC06 supplement groups was significantly up-regulated, indicating that BaSC06 could promote the polarization of macrophages toward the M2 phenotype by activating the STAT3 signaling pathway. *In vitro*, Shiraishi et al. observed that glucagon-like peptides-1 can up-regulate the expression of phosphorylated STAT3 protein and activate STAT3 to mediate macrophage polarization toward the M2 phenotype (86), which is consistent with our results.

Maintaining intestinal microorganisms balance is essential for intestinal development and homeostasis, which further resists pathogenic bacteria more effectively. Intestinal microorganisms are mainly classified into about 50 phyla. Changes in the composition of the intestinal microbiota affect animal health, such that we can improve animal health by changing the composition of their intestinal microbiota. For example, adding Holly polyphenols in pigs diet alleviated LPS-triggered intestinal injury by altering microbiota composition while reducing intestinal inflammation (87). In the human intestine, *Firmicutes* and *Bacteroidetes* are dominant, while *Proteobacteria, Actinobacteria, Clostridia* etc. account for only a few (88). We found that *Firmicutes* and *Bacteroidetes* to be the dominant bacteria in the cecum content of pigs for fattening. The spherical or rod-shaped *Firmicutes* mainly comprise gram-positive bacteria with low G+C content. The most important classes including *Bacillus, Enterococcus, Lactobacillus, Lactococcus*, and other beneficial bacteria, which play an important role in maintaining animal gut health (89). *Bacteroidetes* are Gram-negative bacteria and the second-largest bacterium found in the intestines of healthy people or animals. *Proteobacteria* are also Gram-negative bacteria, which is the largest proportion of the bacteria, including many pathogenic bacteria, such as *E. coli, Salmonella, Vibrio, Helicobacter*, etc. The beneficial effects of probiotics may come from inhibiting the growth of pathogenic bacteria and promoting the growth of beneficial flora in the gastrointestinal tract. Probiotic colonization and its effect on members of the intestinal microbiota are highly species-specific. *Enterococcus faecium* and *Lactobacillus* can reduce the abundance of *E. coli* and increase the abundance of anaerobic bacteria in the ileum and cecum of piglets (90). Together with other anaerobic microorganisms, *Bacillus* may occupy the surface of the intestinal mucosa to create a biological barrier to protect the intestinal mucosa and prevent the invasion of pathogenic bacteria. In addition, they can inhibit the growth and reproduction of harmful microorganisms such as *Clostridium*, thereby improving the disease resistance and growth performance of piglets (91, 92). *Bacillus* increased the abundance of *Bacillus, Bifidobacterium*, and *Lactobacillus* in the gut of pigs, while relatively reducing the abundance of *E. coli* (93–96). This study found that BaSC06 did not affect the diversity of the intestinal microorganisms of the pigs for fattening compared to Anti group. However, it could improve the microbiota composition, especially by significantly increasing the proportion of Gram-positive bacteria (*Firmicutes*), and reducing the proportion of gram-negative bacteria (*Bacteroidetes, Proteobacteria*). Therefore, the probiotic BaSC06 could promote the balance of the intestinal microorganisms, improve the microbial barrier function, strengthen the dominant position of the *Firmicutes*, and significantly reduce gram-negative pathogen infection, thereby promoting the intestinal health of pigs for fattening.

## Conclusions

In summary, oral administration of BaSC06 instead of antibiotics improves the growth of pigs for fattening via increasing the activity of intestinal enzymes involved in digestion and absorption, and up-regulating the expression of transporters. It also enhances antioxidant capacity by activating the Nrf2 signaling pathway and decreasing ROS production. In addition, it improves intestinal integrity, intestinal immune function and trigger M1 and M2 macrophage polarization via STAT3 signaling pathway, as well as improves the composition of intestinal microbiota. Hence, *Bacillus amyloliquefaciens SC06* appears to be an effective feed supplement to antibiotics in piglets and pigs for fattening. However, further research on BaSC06 should be performed to determine its impacts on intestinal development, repair of injured intestinal and stem cell differentiation.

## Data Availability Statement

The datasets presented in this study can be found in online repositories. The names of the repository/repositories and accession number(s) can be found below: NCBI SRA (PRJNA664482).

## Ethics Statement

The animal study was reviewed and approved by the Institutional Animal Care and Use Committee of Zhejiang University.

## Author Contributions

WL and XC conceived and designed the experiments. XC, LT, and ZZ conducted the experiments. XC, LT, and BW analyzed the data and made the figures. LT wrote the manuscript. WL, LT, XC, YZ, QW, and PZ revised the manuscript. All the authors reviewed and approved the final version of the manuscript and agreed to be accountable for the content of the work.

## Conflict of Interest

The authors declare that the research was conducted in the absence of any commercial or financial relationships that could be construed as a potential conflict of interest.

## Abbreviations

BaSC06: *Bacillus amyloliquefaciens SC06*
ADFI: Average daily feed intake
ADG: Average daily gain
AKPase: Alkaline phosphatase
Arg: Arginase
ASCT: ASC-type animo acid transporter
B^0^AT1: B^0^-type amino acid transporter 1
CAT: Catalase
EAAC1: Excitatory amino acid carrier 1
F/G: Feed/gain
GAPDH: Glyceraldehyde-3-phosphate dehydrogenase
GLUT2: Glucose transporter 2
GSH: Glutathione
GSH-Px: Glutathione peroxidase
GST: Glutathione S-transferase
HO-1: Hemeoxygenase-1
HRP: Horseradish peroxidase
IL: Interleukin
iNOS: Inducible nitric oxide synthase
IPEC: Intestinal porcine epithelial cells
IRF3: IFN-regulatory factor 3
Keap1: Kelch-like epichlorohydrin-associated protein 1
LAT1: L-type amino acid transporter 1
LPS: Lipopolysaccharide
MAPK: Mitogen-activated protein kinase
MCP1: Monocyte chemotactic protein 1
MDA: Methane dicarboxylic aldehyde
MR: Mannose receptor
Na^+^, K^+^-ATPase: Sodium potassium ATPase
NOX: NADPH oxidase
NQO1: NAD (P)H: Quinone oxidoreductase 1
Nrf2: Nuclear transcription factor-erythroid 2-related factor
PEPT1: Peptide transporter 1
SGLT1: Sodium-dependent glucose transporters 1
STAT: Signal transducer and activator of transcription
T-AOC: Total antioxidant capacity
TNF-α: Tumor necrosis factor-α
T-SOD: Total superoxide dismutase
V/C: The height of the villi/the depth of the crypt
γ-GT: γ-glutamyl transpeptidase.
