# Supplementary material for: Effects of Probiotics BaSC06 on Intestinal Digestion and Absorption, Antioxidant Capacity, Microbiota Composition, and Macrophage Polarization in Pigs for Fattening
Source: Front Vet Sci. 2020 Nov 6;7:570593. doi: 10.3389/fvets.2020.570593 (PMC7677304; doi:10.3389/fvets.2020.570593)
Supplement: Supplementary file 1 [file Table_1.doc]

**Table. S1 List of real-time PCR primers**

| Gene | Acc. No | Sequence of primers | Length |
| --- | --- | --- | --- |
| GAPDH | NM_001206359.1 | F：GGTCGGAGTGAACGGATTT | 245 |
|  |  | R：ATTTGATGTTGGCGGGAT |  |
| SGLT1 | NM_001164021.1 | F：GTGATTTATACGGATACCT | 171 |
|  |  | R：ATAACATTCTTTCTTGATGG |  |
| GLUT2 | NM_001097417.1 | F：AGGCATATCAGGACTCTACT | 77 |
|  |  | R：ACTTGGTTGGAGCAATCT |  |
| PEPT1 | NM_214347.1 | F：CAACATCATCGTGCTTATC | 171 |
|  |  | R：TCGTCCATATCAAACTGAG |  |
| B^0^AT1 | DQ231579.1 | F：CTGTCCACAACAACTGCGAGAA | 329 |
|  |  | R：GAAGACGATGAAAGCCAACCC |  |
| ASCT2 | DQ231578.1 | F：TGGTCTCCTGGATCATGTGGT | 203 |
|  |  | R：GAAGCGGTAGGGGTTTTTGC |  |
| EAAC1 | NM_001164649.1 | F：ATCGTTCAAATCATTATGTG | 167 |
|  |  | R：TATATCAGTGGCAGAATAAC |  |
| LAT1 | EU390782.2 | F：GGAACTGGGCACCACCATTA | 161 |
|  |  | R：GCACCACGTAGTTGGCAAAG |  |
| GST | AB000884.1 | F：CAACCCAGAAGACTGCTCAAG | 159 |
|  |  | R：GGACCACTCAAGGAATACAGAAG |  |
| HO-1 | NM_001004027.1 | F：AGCTGTTTCTGAGCCTCCAA | 130 |
|  |  | R：CAAGACGGAAACACGAGACA |  |
| NQO1 | NM_001159613.1 | F：CCAGCAGCCCGGCCAATCTG | 160 |
|  |  | R：AGGTCCGACACGGCGACCTC |  |
